# A Rare Case of Intracranial Nongerminomatous Germ Cell Tumor in a 21-Year-Old Romanian Male

**DOI:** 10.1155/2020/3787250

**Published:** 2020-01-03

**Authors:** Minesh Nandi, Rahul Anil, Edward Hamaty, William Adams, David Stidd, Krizelle Garde, Hari Kandukuri

**Affiliations:** ^1^Department of Neurological Critical Care Medicine, AtlantiCare Regional Medical Center, 1925 Pacific Avenue, Atlantic City, NJ, USA; ^2^Medical Neurological Critical Care, AtlantiCare Regional Medical Center, 1925 Pacific Avenue, Atlantic City, NJ, USA; ^3^Department of Neurosurgery, AtlantiCare Regional Medical Center, Atlantic City, NJ, USA; ^4^Department of Neurological Surgery, Thomas Jefferson University and Jefferson Hospital for Neuroscience, Philadelphia, PA, USA

## Abstract

Extragonadal germ cell tumors are a rare entity that is more prevalent in infants and young children, with preference to midline structures. The category of intracranial germ cell tumors is divided into pure germ cell tumors (GCTs) versus nongerminomatous germ cell tumors (NGGCTs). They are usually present in the second decade of life with a male preponderance. We present here a rare case of intracranial NGGCT in a 21-year-old Romanian male, who presented with complaints of emesis, ataxic gait, and diplopia. A computed tomography scan of the head in the emergency department revealed a pineal/suprapineal mass along with obstructive hydrocephalus and dilated lateral and third ventricles without any bleeding. MRI of the cervical, thoracic, and lumbar spine showed no evidence of leptomeningeal metastasis. The patient had elevated serum markers of beta-hCG and AFP, which pointed towards a diagnosis of nongerm cell tumor, as in pure GCTs, these markers are normal. To relieve the obstruction from the mass effect, the patient had an endoscopic third ventriculostomy (EVT). However, after the procedure, he developed central diabetes insipidus as a complication with a triphasic response. Biopsy of the mass revealed atypical cells with granular architecture and atypical glands with positive immune histological markers for NGGCT. These findings supported the diagnosis of mixed germ cell tumor with yolk sac carcinoma and seminoma components. Patient's transient central diabetes resolved with normalization in his urine output. He was eventually stabilized and returned to Romania for further management. In summary, intracranial germ cell tumors are rare brain tumors that should be distinguished based on histology and tumor markers as they will help in the guidance of therapy. An initial evaluation with neuroimaging, tumor markers, cytology from CSF, and biopsy is a must to distinguish further treatment and prognosis.

## 1. Introduction

Germ cell tumors (GCTs) can be gonadal or extragonadal depending upon the evidence of the existence of a primary tumor in the testis or ovaries [1]. GCTs are classified extragonadal if there is no evidence of primary tumor in the gonads. Extragonadal germ cell tumor typically arises in locations that are midline, including anterior mediastinum, retroperitoneum, pineal gland, and suprasellar regions [1]. Distribution is unimodal, with a sudden surge in frequency in early pubertal years diagnosed between ages 10 and 21 years with a male preponderance ratio of 3 : 1 [1]. In North America, intracranial germ cell tumor represents 0.5-3% of pediatric central nervous system tumors [2]. Patient's clinical presentation varies by location and size including visual changes and signs of increased intracranial pressure [1]. Neuroimaging techniques are useful in detecting the site; however, one cannot differentiate GCT types based on imaging alone, as a diagnosis usually requires histologic confirmation. The molecular pathogenesis of intracranial GCT gain of chromosome 12p which is considered characteristic for germ cell tumor of the testes was observed [3]. No differences were found in genetic profiles of GCT versus NGGCT on comparative genomic hybridization studies; however, average imbalances occurred in the latter group [3].

A meta-analysis comparing gonadal and extragonadal germ cell tumors revealed virtually no difference in genomic alterations in the tumors [3]. As per histologic classification, approximately 25% of NGGCT are mixed, and they contain more than one histologic component. These can include yolk sac, endodermal sinus, and syncytiotrophoblast components. While parasellar tumors present with diabetes insipidus and hypothalamic-pituitary axis dysfunction, the NGGCTs usually present as posterior third ventricular masses with obstructive hydrocephalus. Intracranial GCTs are divided further based on tumor markers in CSF and serum. These include alpha-fetoprotein (AFP) and beta human chorionic gonadotropin (b-hCG) with immunohistochemical markers including alkaline phosphatase and c-kit on the tumor cells [4].

## 2. Case Presentation

We present here a case of a 21-year-old right-handed Romanian male, who was working in the United States on an employer-sponsored visa and presented to the emergency department of Atlantic Regional Medical Center in Atlantic City, New Jersey, with acute episodes of vomiting, diplopia, and ataxic gait and two episodes of tonic-clonic seizures. The patient was brought in by his acquaintances who reported headaches for two weeks along with vision changes and a slow decline in his mentation. His friends stated that the patient was becoming more tired and lethargic with a waxing and waning in mentation. Pertinent physical exam revealed Parinaud's syndrome with upward gaze palsy, diplopia, and unsteady gait. A CT scan was done in the emergency department that showed a pineal mass of 3 × 3 × 3.5 mm in size along with obstructive hydrocephalus and dilatation of lateral and third ventricles without any evidence of bleeding. The patient also had an episode of seizure in the emergency department and was administered a loading dose of Keppra 1000 mg and a maintenance dose of 500 mg twice a day intravenously. The patient was then admitted to the neurological critical care unit for further monitoring.

**Figure 1:**
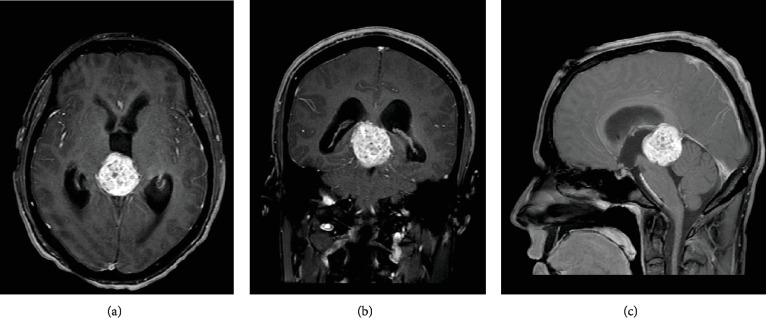
(a) Axial, (b) sagittal, and (c) coronal view of the intracranial germinoma. Note the obstructive pattern of the tumor giving obstructive hydrocephalus.

**Figure 2:**
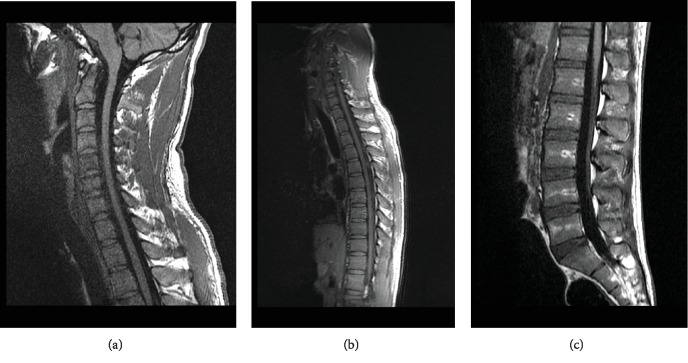
MRI of the cervical (a), thoracic (b), and lumbar spine (c) showing no evidence of leptomeningeal spread.

**Figure 3:**
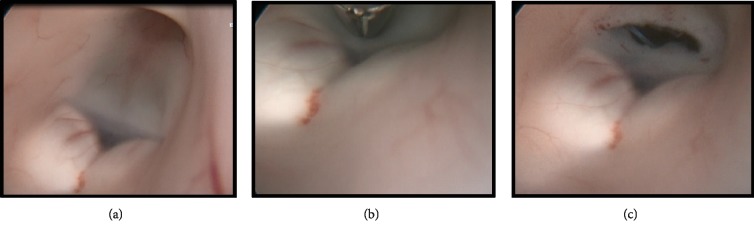
The image shows the endoscopic third ventriculostomy (EVT) procedure.

Neurosurgery evaluated the patient and recommended to bolus 10 mg of IV dexamethasone and with the maintenance of 4 mg IV every 6 hours to prevent expansion and reduction of the cytotoxic edema from the mass effect. Given his symptoms of obstructive hydrocephalus with increased ICP of 22 mmHg, an endoscopic ventricular drain was placed for the drainage to relieve the pressure by the neurosurgeon. MRI of the brain revealed a pineal mass causing obstructive hydrocephalus via the compression of the cerebral aqueduct likely from a possible underlying intracranial germ cell tumor (Figure 1, image R to L). MRI of the cervical, thoracic, and lumbar spine was done with and without contrast. No enhancing lesions were visualized on these images, and there was no evidence of drop metastasis or discrete mass lesion or pathologic enhancement in the spinal system (Figure 2). A detailed MRI of the lesion revealed mass arising from the pineal gland with a diameter of 3.6 cm and predominantly solid component with discrete calcification inside the mass (Figures 1(a)–1(c)). The tumor was causing compression of the tectal plate of the midbrain and subjacent aqueduct, which was resulting in obstructive hydrocephalus. To further evaluate, we obtained serum beta-hCG and alpha-fetoprotein to distinguish if the mass was a pure extragonadal germ cell tumor or NGGCT. Patient's serum alpha-fetoprotein levels were elevated to 215.8 ng/mL (normal range of 0-8 ng/mL), and beta-hCG level was noted to be 35 mIU/mL (normal range of 0-3 mIU/mL). Given the elevations of the tumor markers, this patient likely had an NGGCT; however, a definite diagnosis biopsy was needed. On day three of his admission, the patient received an endoscopic third ventriculostomy to improve the obstructive hydrocephalus and help in the drainage of the cerebrospinal fluid through the cerebral aqueduct system during which we also obtained a biopsy of the sample (Figure 3).

However, after this procedure, the patient developed a transient episode of central diabetes insipidus. On his laboratory analysis, he was noted to have sodium level of 145 mEq/L (range 135 mEq/L-145 mEq/L) with a urine osmolality of 161 and serum osmolality of 298. Central diabetes insipidus is defined as serum sodium concentration higher than 142 mEq/L in a setting of polyuria of 3000 cc/day with a plasma osmolality of 295 and a low urine osmolality, which were all noted in this patient. This was a complication likely from the EVT procedure. The patient was observed to be in transient triphasic response with the polyuric phase for five days, followed by an antidiuretic phase of 6 more days. We monitored his urine output very closely replacing cc per cc of volume by giving him by mouth and isotonic IV fluids. The patient was started on desmopressin, which was administered in the intravenous form initially and then converted to intranasal.

**Figure 4:**
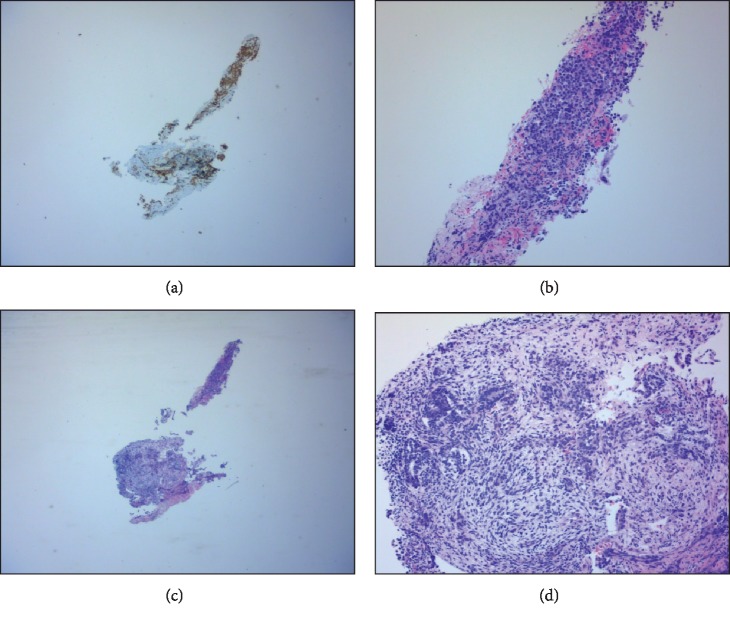
(a) Immunohistochemical stain of SALL-4, a marker for germ cell tumor. (b) Seminoma portion, 100x. (c) H&E of the whole biopsy. (d) Yolk sac carcinoma, 100x. Note the glands with atypical cells forming glandular architecture and solid area, which are all positive for Sal-4 and negative for CD30 and GFAP (glial fibrillary acidic protein). The atypical glands are positive for pancytokeratin and glypican-3, and the solid area is positive for OCT3/4 and CK117. These findings supported a diagnosis of mixed germ cell tumor with yolk sac carcinoma.

The patient subsequently developed hyponatremia likely also contributed from the desmopressin, and he was noted to develop cerebral salt wasting for which he was placed on hydrocortisone and later changed to fludrocortisone. Patient's urine output decreased at a goal less than 3 L in 24 hours with a normal serum osmolality. Pathology report came back, which revealed atypical cells were forming glandular architecture and solid area, which are all positive for Sal-4 and negative for CD30 and GFAP (glial fibrillary acidic protein) (Figure 3). The atypical glands are positive for pancytokeratin and glypican-3, and the solid area is positive for OCT3/4 and CK117 (Figure 4). These findings supported a diagnosis of mixed germ cell tumor with yolk sac carcinoma. However, due to the limited material, other components cannot be ruled out. Patient's electrolytes were normalized, and he was medically cleared to travel back to Romania where he would be following up with neurosurgery for further management of his NGGCT.

## 3. Discussion

This case represents an acute presentation with challenging complications in the management of location-sensitive intracranial germ cell tumor. The patient presented with the symptoms of pineal mass, including diplopia, ataxia, headaches, and vomiting, all which are signs of increased intracranial pressure. Patient's neuroimaging revealed a picture of obstructive hydrocephalus. Given the CT scan and MRI findings (Figures 1 and 2), prompt neurosurgical intervention was initiated, and the patient received an endoscopic ventricular drain to relieve his obstruction and decrease the intracranial pressure. Given his clinical signs of increased intracranial pressure, imaging is a must. We did not perform a lumbar puncture, and of note in cases such as this, lumbar puncture should be avoided in these patients due to risk of herniation. Serum alpha-fetoprotein and beta-hCG levels were elevated, pointing more towards the diagnosis of mixed germ cell tumor or immature teratoma. For diagnosis and staging, a histologic examination is needed to establish a definitive diagnosis of an intracranial GCT. Surgery to obtain specimen is mandatory for patients with normal CSF, serum alpha-fetoprotein, and beta-hCG as pure germ cell tumor or mature teratoma must be distinguished from other lesions since the therapeutic approaches are different.

Regarding imaging, MRI is preferred for diagnosis and staging, although CT is sensitive in detecting the lesions, as shown in Figure 1. During the evaluation of a sellar mass, it is imperative to obtain hormonal evaluation including serum prolactin, insulin-like growth factor, and 24-hour urinary free cortisol for lactotroph, somatotroph, or corticotroph adenomas. On MRI, the tumors appear isointense or hypointense on T1 sequences and hyperintense on T2 sequences. The tumor has homogeneous enhancement if no cysts are present or heterogeneous in appearance with the presence of cysts (Figure 1). As noted, our patient has a heterogeneous MRI appearance of his mass with cystic enhancements. MRI of the entire spine is also necessary for the staging of intracranial GCTs as 15% of patients will have leptomeningeal spread [1, 5].

Given the above-discussed markers, any tumors with elevated AFP (>10 *μ*g/L) can contain elements of endodermal sinus tumor or a mature teratoma. Hence, if surgery is not possible for confirmation of diagnosis, such masses should be treated as having NGGCT. Serum AFP > 1000 *μ*g/L has been identified as a poor prognostic indicator [6]. However, a significant portion of these tumors have mixed components, and hence, tumor markers should not be used for risk stratification without an official tissue diagnosis. AFP and beta-hCG from CSF are more sensitive than serum, and both should be obtained in the absence of clinical contraindication. A lumbar puncture (LP), if not contraindicated, is more accurate for tumor markers and then ventricular CSF; however, if LP is contraindicated due to increased ICP, then tumor markers from ventricular CSF and serum can be used for diagnostic purposes. A biopsy is challenging as the pineal gland is a deep structure surrounded by essential vasculature and is associated with complications. Immediate neurosurgical intervention is indicated for obstructive hydrocephalus from a pineal mass or acute visual deterioration from a suprasellar mass. As a result, our patient received an endoscopic third ventriculotomy procedure to relieve his obstruction (Figure 3). Surgical biopsies can yield small samples which can lead to inaccurate diagnosis depending on the area where the tissue was obtained from as mixed tumors and contain various components, and a small area biopsy may only include one part. As a result, when tissue diagnosis is not reliable, treatment should be based upon the outcome associated with most malignant histology and worse prognosis.

In such cases, for example, a tissue diagnosis of NGGCT with normal AFP or beta-hCG levels should be treated as such and not a pure germ cell tumor. Given the location of the tumor along with surgical complication, a gross resection of the mass is generally not recommended. As emphasized, before the distinction of a true germinoma from NGGCT is crucial. Pure germ cell tumors are sensitive to radiation therapy with long-term progression-free survival rates greater than 90% in those patients after radiation therapy [7]. In a study with 48 patients that were confirmed by histology to have a primary CNS germ cell tumor, it was revealed that treatment with radiation with doses higher than 40 Gy to the primary tumor was associated with better control [7]. Prior treatment of the localized germinoma included receiving craniospinal irradiation CSI 36 Gy with a boost to the primary tumor of 50 Gy; however, studies demonstrated that with whole-brain or whole-ventricle irradiation resulted in spinal failure rates less than 10% [7].

As a result, the current standard of care for radiation alone for localized germinoma is 21-24 Gy to the whole ventricle with the boost to the tumor for a total dose of 40-40 5 Gy. Due to the prevalence of NGGCT much less common than pure germinoma, there are no significant studies available to interpret the treatment. NGGCTs are mostly not sensitive to radiation with a small series study showing patient treated alone with radiation therapy, and craniospinal irradiation with a boost to the local tumor site had a survival rate of 20-40% [8]. As a result, in the absence of randomized studies, the current standard of care for patients with intracranial NGGCTs is neoadjuvant chemotherapy, followed by craniospinal irradiation. The tumors are more sensitive to a platinum-based compound, including cisplatin or carboplatin along with etoposide [9]. Studies had shown that neoadjuvant therapy before initiation of radiation therapy resulted in long-term survival (60-70% of cases) [9]. In SIOP CNS GCT study, the magnitude of alpha-fetoprotein elevation was correlated with adverse prognostic indicator with AFP > 1000 U/L revealing a progression-free survival rate of 32% versus 76% in those with AFP < 1000 U/L [5]. Another potential prognostic indicator is the presence of residual disease upon completion of neoadjuvant chemotherapy. In the SIOP CNS 96 trial, the progression-free survival was 85% for dose with no residual tumors versus 48% for those with residual tumors [5]. Also, in patients with recurrent NGGCTs, prognosis is poor.
